# A Multifaceted Explanation of the Predisposition to Buy Organic Food

**DOI:** 10.3390/foods9020197

**Published:** 2020-02-15

**Authors:** Francisco Sarabia-Andreu, Francisco J. Sarabia-Sánchez, María Concepción Parra-Meroño, Pablo Moreno-Albaladejo

**Affiliations:** 1 Social Sciences, Law and Business Administration Department, Catholic University of Murcia (UCAM), Campus de Los Jeronimos, Guadalupe, 30107 Murcia, Spain; mcparra@ucam.edu; 2 Department of Business and Financial Studies, Miguel Hernandez University, 03202 Elche, Spain; fransarabia@umh.es; 3 Department of Marketing and Market Research, University of Murcia, 30100 Murcia, Spain; p.moreno@um.es

**Keywords:** implicit attitudes, explicit attitudes, hedonic dimension, utilitarian dimension, organic food, implicit association test

## Abstract

This study explores whether implicit and explicit attitudes toward organic products explain consumers’ predisposition to buy organic food, considering the hedonic and utilitarian dimensions of attitudes. The data are from an online survey, which included a section on implicit attitudes (measured using an Implicit Association Test) and a section on explicit attitudes. Two products were analyzed using 557 responses from a panel of consumers: chocolate (hedonic-oriented food) and milk (a utilitarian-oriented food). Confirmatory factor analysis and multigroup structural equations were applied to assess the proposed model. Three findings may be highlighted. First, in the model with the lowest entropy, the hedonic and utilitarian dimensions are considered to be independent. Second, different types of attitudes play different roles depending on the product. Finally, implicit attitudes influence the predisposition to buy organic food in the case of pleasure-seeking food but not utilitarian-oriented food. Thus, there is convergence between implicit and explicit attitudes for hedonic-oriented foods and divergence between such attitudes for utilitarian-oriented foods. This study’s value lies in the novel use of implicit attitudes, which have generally been neglected in attitudinal models in the organic food domain.

## 1. Introduction

Consumers increasingly buy organic food because of its perceived superiority to conventional food [1,2]. The general opinion is that organic food is healthier, more nutritious, and more natural than conventional food. This view, along with organic food’s greater perceived environmental friendliness, has led many consumers, institutions, and producers to adopt a pro-organic stance. Nevertheless, there is literature that has expressed doubts over these benefits, questioning whether organic food really is better than conventional food [3], and whether it really does have less of an impact on the environment [4]. In fact, it has been argued that food labeled as organic “does not say anything directly about the product, only about the production method” [5]. However, consumers believe that organic food is synonymous with environmental friendliness and naturalness [6], and the huge growth in demand for organic food shows that consumers perceive buying organic food as a positive choice.

Consumers’ decisions to buy organic food go beyond the evaluation of purely objective and conscious criteria [7]. These decisions are instead shaped by psychological precursors. One of the most studied precursors is attitude. Many studies have addressed attitudes as a key element in the development of behavioral intentions and subsequent buying. However, most studies have focused on only one type of attitude, namely explicit attitudes, because it is the consumers themselves who report these attitudes. It is argued [8] that attitudes lie in people’s choice processes, regardless of whether they are consciously perceived, implying the existence of implicit attitudes. Besides a few recent studies [9,10,11], research in the food domain has paid scant attention to the role played simultaneously by implicit and explicit attitudes.

The literature also reports that attitudes are not unidimensional; they have both a hedonic dimension and a utilitarian dimension [12,13]. These two dimensions, which are intrinsic motivations that shape predispositions to buy, are fundamental to understand how consumers develop their attitudes. In food, the utilitarian dimension refers to aspects related to nutritional goodness and/or the enhancement of their organoleptic characteristics. In contrast, the hedonic dimension relates to features such as the pleasure of consumption or the search for experiences. Thus, consumers may display distinct types of attitudes toward organic foods when different attitudinal dimensions motivate their choices.

Given this background, this study explores the role of implicit and explicit attitudes toward organic food in explaining the predisposition to buy organic food, considering the hedonic and utilitarian dimensions. This paper has four further sections. The following section offers an overview of attitudes (differentiating between implicit and explicit attitudes) and their hedonic and utilitarian dimensions. Hypotheses are also proposed. The next section then outlines the method (measurement instruments and data collection). The penultimate section presents the quantitative results, including validation of the model and hypothesis testing. The paper concludes with a discussion of the findings, limitations, and recommendations arising from the study.

## 2. Literature Review

### 2.1. General Background

#### 2.1.1. Attitudes: Definition, Types, and Model

Attitudes are fundamental precursors of behavior and are crucial to understand the decision and change processes in which personal and social aspects play relevant roles. An attitude can be defined as a natural predisposition or tendency of individuals toward some issue, object, person, or action, which expresses a predilection or dislike toward what is perceived. Eagly and Chaiken define attitude as “an individual’s propensity to evaluate a particular entity with some degree of favorability or unfavorability” [14], Ajzen and Fishbein [15] note that there are two types of attitudes. The first type refers to attitudes toward issues or objects (e.g., organic food) as overall cognitive and emotional interpretations of stimuli. These attitudes tend to be passive, and they largely define the perceptions that individuals have. They can be conscious or unconscious evaluative judgments that are built on any information individuals receive and that determine favorable, unfavorable, or ambivalent opinions.

The second type refers to active attitudes because they are focused not only on a particular phenomenon or object but also on a specific behavior with respect to that issue (e.g., buying organic food). These attitudes are evaluative predispositions that are activated when consumers must choose from the different types of products available. Whereas some choices follow deliberative processes, others follow automatic or impulsive processes.

Both types of attitudes are considered stable predispositions of consumers [16]. They can also be viewed as the result of a subjective comparison of the perceived value of an offering, and it represents a certain buyer’s preference for a particular type of product [17]. Although, from a constructivist perspective, evaluative predispositions are not stored in the mind, studies have shown that attitudes are an efficient strategy for organizing knowledge and making evaluative judgments [18].

The consumer behavior literature highlights three prominent approaches to understanding and measuring attitudes: the multi-attribute model, the affective-cognitive-behavioral (ABC) model, and the Motivation and Opportunity as DEterminants (MODE) model. The multi-attribute model depicts attitudes as consisting of smaller components such as individual object features, functions, or perceived benefits. The ABC model describes attitudes as having affective, cognitive, and behavioral components. Finally, the MODE model [19] considers implicit and explicit attitudes. In the MODE model, implicit attitudes refer to automatic evaluative reactions, whereas explicit attitudes are those that operate on a conscious level. The MODE model is chosen for this study because it includes consideration of implicit attitudes, which is often excluded in other attitudinal models, and bridges a gap in the food literature.

#### 2.1.2. Explicit and Implicit Attitudes

Most psychological theories consider attitudes as a key element to explain who, how, when, where, and/or why individuals behave as they do. Some of these theories only use explicit attitudes, which are formed following a deliberate thinking process and are consciously acknowledged as rational by the individual [15]. However, the existence of a dissonance between actual behavior and declared attitudes may be partly explained by the existence of other attitudes that occur at an unconscious level [8] or that do not emerge because they are deliberately repressed by social conventions [20]. These attitudes, called implicit attitudes, are defined as evaluations of attitudinal objects that are of unknown origin, that are formed using experiences, that are activated automatically, and that influence involuntary responses by individuals [8]. These attitudes are stable and knowable [21].

These two types of attitudes seem to respond to different brain activity. More specifically, the dorsolateral prefrontal cortex is involved in the regulation of implicit attitudes, the amygdala is implicated in the automatic assessment of social stimuli, the prefrontal cortex plays a prominent role in the regulation of explicit attitudes, and, in cases of conflict, specific areas related to the cognitive control of the prefrontal cortex are activated [22]. From a psychologic perspective, explicit attitudes are more closely involved in generating controlled and deliberate evaluations, whereas implicit attitudes are more involved in the processing of automatic and spontaneous events [23]. However, the two processes need not be exclusive: When the brain receives a stimulus, it responds by first activating automatic responses and then providing conscious cognitive responses [24]. In short, individuals tend to process stimuli explicitly, but the information that is received also generates implicit attitudes through unconscious or preconscious associations [25,26].

In relation to food, implicit attitudes are associated with spontaneous behaviors such as buying healthy food [27], explicit attitudes predominate in deliberative behavior [9,28,29], and both play relevant roles in various types of decisions [30,31]. In conclusion, both attitudes are important, although explicit attitudes have a greater influence when consumers show greater self-control motivation, and implicit attitudes predominate in situations of impulsive consumption or low self- control motivation [32]. Since the works of Nobel Prize winner Daniel Kahneman, it has been accepted that there is an automatic (implicit) system of action and another mediated and deliberative (and therefore explicit) system, and these two systems interact in a dual process [33].

#### 2.1.3. Utilitarian and Hedonic Dimensions of Attitudes

Traditionally, the study of attitudes focused on only one dimension [13]. In other words, attitude was interpreted as a continuum with two extremes. However, the theoretical development of the hedonic and utilitarian dimensions in various areas of knowledge changed the way attitudes are understood. Thus, the comprehension of attitudes has evolved from a one-dimensional concept toward a more complex concept [34,35] based on a two-dimensional approach.

Differentiating between the hedonic and utilitarian dimensions of attitudes helps explain consumers’ food preferences and patterns in consumers’ buying decisions [36,37]. Although these dimensions underpin attitudes, the most prevalent dimension depends on the type of food. For example, the utilitarian dimension is prevalent when rational, objective, and sensible criteria are the main buying drivers. Thus, the choice of utilitarian-oriented food depends on fulfilling a practical purpose and achieving an instrumental goal [38]. With some foods, the utilitarian dimension dominates because the relevant attributes for consumers (nutritional value, calorie content, etc.) mean that the choice is aimed at meeting useful objectives (sticking to a diet, losing weight, etc.). Meanwhile, food has a prevalent hedonic dimension when aspects related to pleasure-seeking and other affective reasons are predominant in consumer choices. Accordingly, hedonic-oriented food choices are oriented toward sensory experiences [34,38]. For example, with indulgent desserts such as cakes, sensory features (taste, smell, texture, etc.), which are manifestations of the hedonic dimension of attitudes, may dominate when the consumer’s goal is to feel emotions and pleasure.

Of course, this hedonic versus utilitarian distinction is not exclusive. In certain buying scenarios, products, and decision processes, hedonic and utilitarian dimensions or motives combine [13,39,40]. For example, when buying salt, utilitarian aspects (e.g., preserving and seasoning food) can be combined with hedonic and/or affective aspects (e.g., evocations of place of origin and/or religious norms) that lead consumers to consider different types of salt (e.g., Himalayan pink salt or kosher salt).

### 2.2. Research Model and Hypothesis Development

**Figure 1:**
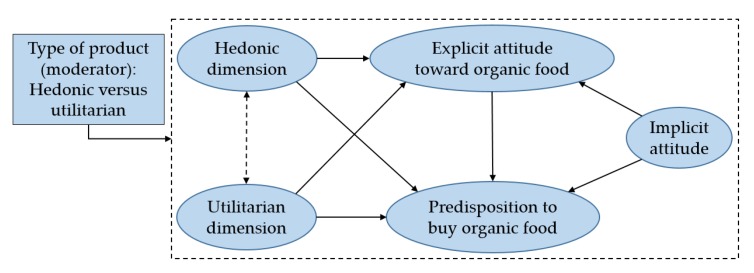
Conceptual model.

The aim of this study is to explore and test whether the aforementioned attitudes can explain consumers’ predisposition to buy organic food, considering the hedonic and utilitarian dimensions of attitudes. Figure 1 shows the overall conceptual model used in this study.

This model can be divided into two sub-models. Model 1 (presented in Figure 1 using solid arrows only) depicts a moderated model of implicit and explicit attitudes, where the hedonic and utilitarian dimensions are independent of one another. It is also possible to consider a second model (Model 2, presented in Figure 1 using the dotted arrow), which includes the possible mutual influence between these attitudinal dimensions.

In relation to these utilitarian and hedonic components of attitudes, the literature is inconclusive. Although most research has shown that these two attitudinal dimensions are independent of one another [41,42], Dhar and Wertenbroch [38] report that utilitarian components are preferential in buying decisions, and Voss et al. [13] note that the two dimensions are mutually related. Other scholars refer to a hierarchy between the two components. For example, Lee and Goudeau [43] affirm that the utilitarian dimension dominates the hedonic dimension when buying organic food, although other researchers [44,45,46] report that food choice is driven by emotional mechanisms and is based on hedonic motives such as pleasure and sensory gratification. Nevertheless, Nasir and Karakaya [47] note that the hedonic dimension of consumer attitudes plays a key role in determining the intention to buy organic food.

Regarding organic foods, Nasir and Karakaya [47], and Lee and Yun [48] report that the utilitarian and hedonic dimensions have a significant impact on consumers’ intentions to buy organic food. However, the possible moderating effect of different product categories has been overlooked in the literature. Moreover, the literature has not examined how perceptions differ between people who tend to buy organic food when considering the hedonic versus utilitarian dimensions of attitudes [48]. Based on these considerations, the following hypothesis may be proposed:

**Hypothesis** **1** **(H1).** *The mutual independence between the utilitarian and hedonic dimensions of attitudes contributes to a better explanation of the predisposition to buy organic food.*

The literature highlights the importance of differentiating between products according to their hedonic or utilitarian nature [49,50,51]. This hedonic or utilitarian nature stems from consumer motivations in relation to the goals that consumers hope to achieve by buying a certain product. More specifically, hedonic-oriented food choices are primarily motivated by consumer experiences and emotional rewards (pleasure, fun, fantasy, etc.). In contrast, utilitarian-oriented food choices are more strongly rooted in rational motivations [52,53] such as nutrition or the pursuit of a rational goal.

Many food choices are shaped by emotional and hedonic aspects rather than rational motivations and functional needs. However, certain foods that are primarily consumed for their utility (e.g., bread and vegetables) may also be subject to emotional motivations. Thus, for some products, the distinction is not exclusive because they might involve a mixture of hedonic and utilitarian aspects [13,39,40], even if one of the two motivational orientations tends to dominate the other when buying the product.

There is extensive research on the motivations that lead to the predominantly hedonic orientation of organic food [47] given its mixture of related aspects such as health, environmental friendliness, and ethics [54]. However, much of the information that consumers receive (from websites, forums, magazines, etc.) stresses the superior attributes of organic food with respect to conventional food. Similarly, in the minds of consumers, there is also a clear difference between food that is oriented toward rational motivations and food that is oriented toward hedonic motivations [55,56,57]. Based on these considerations, the following hypothesis may be proposed:

**Hypothesis** **2** **(H2).** *The type of food (hedonic-oriented or utilitarian-oriented) influences the importance of each type of attitude and attitudinal dimension in explaining the predisposition to buy organic food.*

Figure 1 shows that both implicit and explicit attitudes are precursors to buying. Some scholars note the priority of implicit attitudes over explicit ones [58], although this relationship is still being studied [9] because of the complex attitudinal framework. There is also a broad consensus that implicit and explicit attitudes have a joint influence on behavioral response [32,59,60] and that they compete for the control of individuals’ responses [61]. Thus, implicit attitudes may dominate food choices when individuals make intuitive, automatic choices in impulse buying situations [27] or in situations with restricted cognitive resources [62], whereas explicit attitudes may prevail over implicit ones in, for example, situations of high self-control [32] or situations where choices are binary [9]. In relation to the influence of implicit over explicit attitudes, most of the literature tends to affirm that the two coexist or interact with one other [9] but does not clearly show the priority of implicit over explicit attitudes. However, in this study, this priority is assumed based on the MODE attitudinal model [19], and implicit attitudes are considered predictors of explicit attitudes [27,58].

In the model shown in Figure 1, the utilitarian and hedonic dimensions only influence explicit attitudes (toward organic food or toward buying organic food) because both are established on a stated, conscious level. Based on these considerations, the following hypothesis may be proposed:

**Hypothesis** **3** **(H3).** *For each type of food (hedonic-oriented or utilitarian-oriented) the different types and components of attitudes vary in importance.*

## 3. Method

### 3.1. Participants, Fieldwork, and Data Collection

The target population comprised residents of the United Kingdom aged between 18 and 70 years. Participants were contacted in September 2018 through the consumer panel specialist company Cint, using computer-assisted web interviewing. The researchers had no opportunity to contact the participants because Cint handled the whole process.

Non-random sampling was used. To minimize the usual biases associated with this type of sample, three actions were taken. First, respondents were spread over several cities and areas in the United Kingdom, thereby avoiding sample concentration. Second, the sample was balanced [63] by age and gender. Finally, the fieldwork was controlled [64] during its execution, following the standards established by Cint and the specific instructions given by the authors.

To validate the instruments and perform explanatory analysis of the proposed model, the minimum sample size was set to 10 individuals for each item in the measurement instruments. Thus, the sample size *n* was set to 280 (10 × 28 = 280) and was raised to 300 to comply with the limit imposed after the debugging process.

Each potential participant received an invitation via e-mail. This invitation included a brief description of the study, its phases, and the objective pursued. This fieldwork was performed in two phases:

- Phase 1: Product: Chocolate. Period of fieldwork = 1 week. Effective responses = 300. Implicit Association Test (IAT) and questionnaire with images and text focused on this specific product.
- Phase 2: Product: Milk. Period of fieldwork = 5 days. Effective responses = 299. IAT and questionnaire with images and references focused on this product.

Cint was instructed that one respondent could only participate in one of the two phases, not both.

After all responses had been received, a debugging process was performed in two steps:

- Eleven cases were eliminated because of repeated IP addresses. The IP address was unique for each router and session during the fieldwork. Although this elimination rule might have prevented well-intentioned respondents from participating, it also reduced the possibility of receiving several responses from the same person. The survey software automatically deleted the 11 cases without the researchers’ access to the IP information.
- Thirty-one cases were discarded because of straightlining, which was used as an indicator of poor-quality responses [65].

### 3.2. Instruments

The Appendix A at the end of the document shows the items of all the instruments used in the study, as well as the acronyms assigned to the instruments and their respective items.

#### 3.2.1. Implicit Attitudes (IA)

The IAT, which was originally proposed by Greenwald et al. [66], was used to measure implicit attitudes. This technique reveals the unconscious preferences of subjects through associations between different concepts. The underlying idea is that certain concepts might be more closely linked than others in consumers’ minds. The IAT uses a sequential standard protocol of seven sets of tasks. In each task, participants see stimuli that they must classify as quickly as possible by pressing on the keyboard with their left or right hand (see Table 1).

This protocol presents a series of stimuli that combine words and images to elicit latent predispositions in consumers. In this study, the following stimuli were used:

- Words to describe “positive” concepts (items: better, fantastic, beneficial, good, pleasant, and healthy) and “negative” concepts (items: worse, horrible, harmful, bad, unpleasant, and unhealthy).
- Images of the products with different designs to differentiate between organic and conventional products.

The IAT measures the latency of the mental associations made by individuals. To do so, it measures the time it takes for each individual to assign one of the two opposite options in response to each stimulus that appears on the screen. The less time the individual takes to respond, the more automatic (and therefore the stronger) the association between the option and the stimulus is deemed to be. On the contrary, when individuals take longer to associate a stimulus with a response option, cognitive reasoning is considered to be greater, and the automatic or implicit association is deemed to be weaker. The rationale behind the IAT is that it is easier and faster to associate concepts that are consistent with one’s way of thinking than to make associations with concepts with little coherence.

IAT scores are derived from the differences between the mean time between exposure to the stimulus and participants’ responses. These scores are dimensionless and range from −2 to +2. They are comparable to Cohen’s *d* statistic to measure effect size [67]. In this study, the D measure was applied [68], which was used for both the improved IAT and the subsequent Brief-IAT [69,70]. A positive D-score reflected an association between organic foods and positive attributes and between conventional products and negative attributes. A negative D-score reflected the opposite. IATs have been used to study attitudes as precursors to the choice of organic food [71], the halo effect of organic food [72], and attitudinal responses of consumers to organic wine [73], among other phenomena.

The literature shows that the IAT has good internal consistency (reliability of 0.70 to 0.90) [74] and predictive validity [75,76], offering a method with very high psychometric quality [77]. In the field of food, Richetin et al. [71] showed that it has predictive validity in the case of the choice of fruit versus snacks.

The IAT method is robust to the priming effect. For example, Degner [78] found that instructions prior to consumer responses can modify (increase or decrease) the possible priming effect, and Brunel et al. [79] showed that IATs are not related to explicit reasoning. Moreover, Bruni et al. [80] reported their robustness to framing effects and stimuli valence.

In relation to the order effect produced by the fixed position of the tasks (blocks), those performed first tend to interfere with subsequent tasks [66,70]. This order effect exists, and efforts have been made to eliminate it [69,70]. In the present study, the software used to capture the online responses could not eliminate this effect. However, the phenomenon under analysis was of low emotional risk and did not involve placing individuals in situations of deep personal prejudice. Furthermore, it was considered that this effect tends to be small, both in general [81] and when individuals have little information [82], as is the case with organic products. Therefore, it was concluded that the order effect in this study would tend to be quite small.

#### 3.2.2. Hedonic and Utilitarian Dimensions of Attitudes (HED, UTI)

Attitudes reflect the reasons that lead consumers to use products or services. Consumers search for gratification through affective and hedonic aspects while seeking utility for reasons of use [12]. Based on this premise, Voss et al. [13] developed an instrument to measure the hedonic and utilitarian dimensions of consumer attitude. Their proposal consists of 10 statements with a 7-point semantic differential format, where each attitudinal component (utilitarian and hedonic) has five items. In their original article, Voss et al. [13] reported a composite reliability of 0.92 for both attitudinal dimensions and reported multiple forms of validity. This measure, which was used for this study, has subsequently been shown to have high indicators of reliability and validity [83,84].

For the utilitarian dimension, the items that were used were as follows: (UTI1) Effective/Ineffective, (UTI2) Helpful/Unhelpful, (UTI3) Functional/Not functional, (UTI4) Necessary/Unnecessary, and (UTI5) Practical/Impractical.

For the hedonic dimension, the items that were used were as follows: (HED1) Not fun/Fun, (HED2) Dull/Exciting, (HED3) Not delightful/Delightful, (HED4) Not thrilling/Thrilling, and (HED5) Enjoyable/Unenjoyable.

#### 3.2.3. Explicit Attitudes toward Organic Food (PRO)

In the area of organic food, very diverse measurements have been developed yet not applied beyond their use in seminal articles. The instrument developed by Gil et al. [85] is one of the few that has been widely used, although with disparate results. These authors developed a scale with nine items scored on a 7-point scale ranging from 1 (totally disagree) to 7 (totally agree). Several studies [86,87] have reported its reliability (Cronbach’s alpha > 0.85) and convergent and discriminant validity. This measurement is only focused on organic food, and it does not introduce any comparison with conventional food. The items that were used were as follows: (PRO1) organic products (OP) are healthier, (PRO2) OP are higher quality, (PRO3) OP are a fraud, (PRO4) OP are tastier, (PRO5) OP are worse than conventional food, (PRO6) OP are more expensive, (PRO7) OP are more attractive, (PRO8) OP have no harmful effects, and (PRO9) OP are in fashion.

#### 3.2.4. Predisposition to Buy Organic Food (PRE)

Bravo et al. [88] measured attitudes toward buying organic food as the perceived importance of this type of product. However, when consumers buy food, they choose between the conventional and organic products that are available. This choice is derived from a comparison between the perceived benefits and harms of buying organic products. Therefore, to measure this predisposition, the question raised in the questionnaire was “Buying organic products instead of conventional products is?” Items consisted of eight statements drawn from the proposals of Thøgersen et al. [1] and Berndsen and Van der Pligt [89], presented on 7-point semantic differential scales. Therefore, the instrument forced respondents to give a comparative response. The items that were used were as follows: (PRE1) Harmful/Beneficial, (PRE2) Foolish/Wise, (PRE3) Bad/Good, (PRE4) Unpleasant/Pleasant, (PRE5) Against/For, (PRE6) Unfavorable/Favorable, (PRE7) Negative/Positive, and (PRE8) Unattractive/Attractive.

### 3.3. Procedure

#### 3.3.1. Questionnaire

The online questionnaire had three parts. The first part collected respondents’ informed consent. The only respondents who proceeded to the second part were those who agreed to respond. They also stated that they had no visual problems that might prevent them from quickly seeing the stimuli on the screen or motor diseases such as a hand tremor preventing response to the subsequent IAT. The absence of mobility impairment in hands is essential so that the speed of response reflects the intensity of the association between stimuli and words or images and not the difficulty of physically responding.

The second part consisted of the IAT. The third part consisted of questions on the variables of interest (see Section 3.2). Respondents were informed that they could only respond from a desktop computer. Other terminals (smartphones or tablets) were not allowed because their use might have led to a high number of errors. The digital keys on these devices are so small that delays in response might have occurred because of unintentional movement of fingers on the screen and not because of the actual speed of response.

#### 3.3.2. Products

Two questionnaires with the same instruments were developed for two products: a hedonic-oriented food and a utilitarian-oriented food. Individuals were randomly assigned to one of the two questionnaires. The criteria for deciding which products to consider were foods that (a) had organic and conventional alternatives, (b) would meet different consumption needs, (c) would be well known and easily identified by consumers, (d) would be bought frequently, and (e) would be discriminatory in relation to their usefulness and pleasure-seeking. Accordingly, chocolate was used as a hedonic-oriented food with predominantly pleasure-seeking motivations [52,55], and milk was used as a utilitarian-oriented food because the consumption motivation is nutrition based, and it is part of the basic shopping basket [56,90].

**Figure 2:**
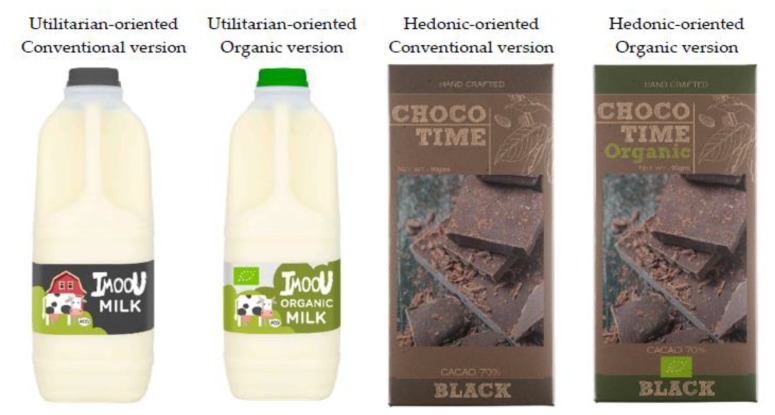
Examples of products designed for the Implicit Association Test.

Figure 2 provides an example of the stimuli used for each product, differentiating between conventional and organic food. The twelve images used in the IAT were fictional and were developed exclusively for this study.

A total of 271 respondents completed the hedonic-oriented food questionnaire, whereas 286 respondents completed the utilitarian-oriented food questionnaire.

### 3.4. Quantitative Methods and Software Used

The IAT was used as the method to measure implicit attitudes. The scores of the IAT were calculated using Greenwald et al.’s improved algorithm [68], and the software developed by Mason et al. [91] was employed to capture online responses.

To check the robustness of the instruments, a confirmatory factor analysis (CFA) was applied, which provides information to calculate the composite reliability (more robust than Cronbach’s alpha) and the convergent and discriminant validities. CFA is a method that is universally used to check whether the items of instruments load on their respective constructs. These constructs are usually obtained previously by means of an exploratory factor analysis or from the analysis of the literature.

Multigroup analysis was applied to check whether the proposed attitudinal model works in the same way for the two types of products, or if the parameters of each specified model do not differ from each other. This statistical technique is a method for testing the differences between the groups, since it forces the measurement and structural coefficients in the two groups to be equal [92]. Regarding the multigroup analysis with moderation, EQS software version 6.1 was used [92].

## 4. Results

### 4.1. Validation of Model Assumptions

This section describes the validation of the instruments that were used, as well as validation of whether consumers perceived the chosen products (milk and chocolate) as different in terms of their utilitarian and hedonic dimensions.

#### 4.1.1. Validation of Instruments

First, principal component analysis is applied to all items of the instruments for the overall sample. This analysis enables description of the latent variables (constructs) and offers clues about their structure. Despite a solution with high values in key statistics (Kaiser-Meyer-Olkin test = 0.92, Bartlett’s sphericity test = 11,578.05, degrees of freedom (df) = 351, probability (*p*) < 0.00), some measures of sample adequacy (MSA) are lower than 0.60. Moreover, the results show that the items of the “attitude toward organic food” construct (denoted as PRO) form three components, which implies a lack of unidimensionality. The MSA scores for items PRO5 and PRO6 are less than 0.60, so they are excluded from further analyses.

Second, because all multi-item constructs are reflective, confirmatory factor analysis (CFA) is conducted to validate the instruments for the overall sample. This analysis excludes implicit attitudes, which is calculated using the D-scores described earlier. The solution has a high goodness of fit but lacks validity for some instruments. The robust Satorra–Bentler estimation method is used given the absence of multivariate normality (Mardia’s test = 287.71 > 3): Satorra–Bentler scaled chi- square (SBSCS) = 883.95 (df = 269, *p* < 0.001), normed chi-square (NCS) = 3.28, Bentler–Bonett non- normed fit index (BBNNFI) = 0.91, comparative fit index (CFI) = 0.93, root mean square error of approximation (RMSEA) = 0.06, 90% confidence interval of RMSEA [0.06, 0.07]. However, the factor loadings (λ) of three items (PRO3, PRO8, and PRO9) are not acceptable due to λ < 0.70. After these items had been eliminated, the solution fit improved slightly: SBSCS = 708.76 (df = 203, *p* < 0.001), NCS = 3.49, BBNNFI = 0.92, CFI = 0.94, RMSEA = 0.07, 90% confidence interval of RMSEA [0.06, 0.07].

In this new solution, convergent and discriminant validities of the instruments are confirmed. Convergent validity is verified because all standardized factor loadings (λ) are greater than 0.60 and significant (*p* < 0.001), as shown in the Appendix A. Discriminant validity is verified because all values of the average variance extracted (AVE) are higher than 0.50 [93]. The confidence intervals of the Pearson’s correlations between the instruments are used to verify discriminant validity. No interval contains a correlation equal to 1 [94,95]. The values obtained for each comparison are shown in Table 2.

#### 4.1.2. Validation of Differences between Chosen Products

To test whether the consumers really perceived milk as a utilitarian-oriented food and chocolate as a hedonic-oriented food, two analyses were carried out using *t* tests and Cohen’s d indicator. First, milk and chocolate were compared to observe whether they differed in their scores. Table 3 shows the results. Regarding the hedonic dimension, chocolate has a significantly higher score than milk (Mean_chocolate_ = 5.88, Mean_milk_ = 4.79, *t* = 11.24, *p* = 0.00, Cohen’s *d* = 0.95). In contrast, in relation to the utilitarian dimension, milk has a significantly higher score (Mean_chocolate_ = 4.94, Mean_milk_ = 5.91, *t* = −10.17, *p* = 0.00, Cohen’s *d* = 0.85).

Because the products were shown to have a defined orientation, tests were also conducted to observe whether consumers perceived each product as inherently utilitarian or hedonic. As expected, the two products have significant differences: For chocolate: Mean_hedonic_ = 5.88, Mean_utilitarian_ = 4.94, *t* = 14.20, *p* = 0.00, Cohen’s *d* = 0.86; for milk: Mean_hedonic_ = 4.79, Mean_utilitarian_ = 5.91, *t* = −16.15, *p* = 0.00, Cohen’s *d* = 0.95.

### 4.2. Descriptive Analyses

#### 4.2.1. Participants

In total, 605 consumers were asked to participate, but 557 valid responses were retained (92.07%). In the valid sample, 48.7% were men, and the average age was 40.9 (standard deviation = 12.74, skewness = 0.13, kurtosis = −0.91). Regarding educational level, 40.4% had compulsory secondary education, 21% had post-16 education, and 38.6% had university studies.

#### 4.2.2. Variables: Values and Distributions of Variables

After the psychometric properties of the instruments had been tested, descriptive statistics for the variables were calculated (Table 4). Implicit attitudes follow a normal distribution (Kolmogorov–Smirnov’s test = 0.02, df = 557, *p* = 0.20), although it is slightly biased toward high values. Accordingly, 48.8% of the sample associates organic food with positive attributes and conventional products with negative attributes, 21.8% does not express a specific association, and 29.4% associates conventional products with positive attributes and organic food with negative attributes. This bias in associating organic food with positive attributes is especially high (D > 0.65) for 13.5% of the sample, whereas only 7.2% strongly associates conventional products with positive attributes.

The hedonic and utilitarian dimensions of attitudes have high average values, although they differ by type of product. Milk has higher values for the utilitarian dimension, and chocolate has higher values for the hedonic dimension, as expected. The respondents also report high values in general when expressing choices, which is indicative of the respondents’ strong attitudes toward organic food.

### 4.3. Hypothesis Testing

To test the first hypothesis (H1), it was necessary to check which model simultaneously had a better fit and was more parsimonious. Therefore, multigroup structural equation analysis was performed. The models shown in Figure 1 were run depending on whether the attitudinal dimensions were taken as independent or as having a mutual influence. Table 5 shows the results.

These results show that both models have adequate levels of fit because all indicators meet the recommended values suggested in the literature [93]. The most parsimonious model was chosen. Neither the Bayesian Information Criterion (BIC) (the restricted model is nested in the unrestricted one) nor Akaike’s Information Criterion (AIC) (both models have the same latent variables and structure) could be used. In this study, the likelihood ratio criterion of the normed chi-square (NCS) based on the Satorra-Bentler Scaled Chi-square (SBSCS) was used because there was no multivariate normality. However, both models had the same NCS ratio (see Table 5). Therefore, we followed Bentler’s criterion, according to which, “simpler models are desired for reasons of parsimony” [92]. Consequently, the best solution was to consider that the hedonic and utilitarian dimensions were mutually independent.

To test the second hypothesis (H2), it was necessary to consider the loading coefficients in the two construct equations (one for chocolate as a hedonic-oriented food and one for milk as a utilitarian-oriented food). Each construct equation represents the construct pattern for one specific product type. Equality between the construct equations implies that the latent pattern is the same (they are from the same population). To check H2, two methods were applied. The first method was based on the chi-square difference between the non-constrained and constrained models. Here, ∆Chi-squared = 33.01, with df = 14 (*p* = 0.00). Thus, it may be concluded that the restrictions lead to a worsening in the fit of the model. The second method was to apply a *t* test to calculate the significance of the difference for each pair of construct loadings [96]. Table 6 shows the coefficients (loadings) of each variable in the two samples, along with the robust standard errors (in parentheses).

Accordingly, H2 may be accepted (the two construct equations respond to different construct patterns) because the restrictions introduced significantly worsen the model fit, and the differences affect most of the model constructs.

To validate Hypothesis 3 (H3), the standardized coefficients of each equation were analyzed because they enabled comparison within the same equation. Table 7 shows this standardized solution. The following findings were observed:

- For chocolate, implicit attitudes (IA), the hedonic dimension of attitudes (HED), and explicit attitudes toward organic food (PRO) are significant, explaining a very high percentage of the predisposition to buy organic food (R^2^ = 0.84). An increase of 1 unit in PRO implies an increase of 0.85 units in PRE, keeping the rest of the variables constant. Among the independent variables, PRO makes the greatest contribution, almost seven times greater than the contribution of IA and almost four times greater than the contribution of the HED.
- For milk, the utilitarian dimension is significant, but the hedonic dimension is not, as reported in the literature [56]. Explicit attitudes toward organic food significantly influence the predisposition to buy organic food. Contrary to the results obtained for chocolate, implicit attitudes are non-significant for the predisposition toward organic food. Here, the explanatory capacity for this product is weaker (*R*^2^ = 0.65), although it is still high. PRO makes the greatest contribution (the increase of 1 unit in PRO implies an increase of 0.78 units in PRE), more than seven times greater than the contribution of the utilitarian dimension of attitudes (UTI). The coefficient of determination is 65.4%. This value is high considering that the equation has only two significant variables. However, 34.6% of the total variance is due to other factors, which is more than half of the value in the case of chocolate (15% of the variance is not explained).

In conclusion, H3 may be accepted because each type and attitudinal component has a different relative importance according to the type of food (hedonic-oriented or utilitarian-oriented).

## 5. Discussion and Implications

This paper explores the influence of implicit and explicit attitudes and the role of the hedonic and utilitarian dimensions on the predisposition to buy organic food. Multigroup structural equation models were run to analyze two independent samples referring to two types of products: chocolate (a hedonic product) and milk (a utilitarian product).

Regarding attitudinal dimensions and the different nature of products, as expected, the influence of each attitudinal dimension varies significantly depending on the nature of the product. For chocolate, the hedonic dimension significantly influences the predisposition to buy organic food. In contrast, for milk, the utilitarian dimension has a significant influence. This finding is consistent with Baltas et al. [55] and Parker et al. [57], who maintain that the utilitarian dimension is not significant for products associated with enjoyment and pleasure, and by Maehle et al. [56] who point out that the utilitarian dimension—not the hedonic one—is significant for utilitarian-oriented foods.

In relation to these attitudinal dimensions, the literature shows that the hedonic and utilitarian dimensions are crucial in the configuration of consumers’ attitudes. This study shows that, at the attitudinal level, both dimensions seem to be independent of one another, so the possible transfer between utility and emotion in consumer evaluations is not considered relevant. This finding is in line with those of Avcilar and Özsoy [41] and Nystrand and Olsen [42] and does not confirm what is reported by Lee and Goudeau [43] and Nasir and Karakaya [47].

With regard to explicit and implicit attitudes, although it has been argued that implicit processes also influence the buying of food [97], the present results reveal two different scenarios: convergence between explicit and implicit attitudes when hedonic-oriented food is under consideration, and divergence in the case of utilitarian-oriented food. Thus, for chocolate (hedonic-oriented food) both types of attitudes influence the predisposition to buy organic food, but they do not do so in the case of milk (utilitarian-oriented food). This scenario of convergence or divergence between the two types of attitudes may be due to a lack of “associative coherence.” In other words, implicit attitudes significantly influence hedonic-oriented foods as they connect better with the attributes of the food. Therefore, with this type of product, cognitive effort is not required to process information. Instead, associations emerge automatically. On the contrary, when dealing with the utilitarian-oriented food (milk), the cognitive elaboration requires attention and reflection. In this case, there is no coherence between the implicit associations and the nature of the product, so implicit attitudes do not have a relevant influence, at least according to the instrument using (IAT).

Regarding the practical implications of implicit and explicit attitudes, it must first be borne in mind that, for food, there is hardly any evidence of the role played by implicit attitudes, and sometimes this evidence is contradictory in relation to the explanatory power of implicit attitudes [98]. This study shows that for different types of products (hedonic-oriented, utilitarian-oriented) implicit attitudes play a different explanatory role. Therefore, it is proposed that future studies differentiate by type of product orientation in order to establish the role played by each type of attitude.

It is well known that implicit attitudes help to discover the automatic underlying feelings that cannot be identified with the classic attitudes stated by consumers [99]. Nevertheless, when analyzing published works on organic food, it is observed that most of the instruments used to measure attitudes are focused on the cognitive aspects of organic foods. In addition, these are treated without focusing on the type of product or whether or not there is a predominance of one or another attitudinal dimension. Therefore, including both dimensions and differentiating between hedonic- and utilitarian-oriented foods can present an opportunity to improve our knowledge about how consumers choose what they buy.

The organic food industry tends to promote hedonic attributes, facilitating the tendency of consumers to make choices based on emotional criteria, which surpass the deliberative aspects (requiring less cognitive effort). Consequently, increasing consistency between product attributes and consumer desires may increase the likelihood that hedonistic individuals will buy organic food because they have a greater attribution of pleasure [100]. In order to define this “pleasurefication” strategy, the natural way would be to transform utilitarian into hedonic attributes. Thus, it would be necessary to know the role played by implicit attitudes, and the analysis of implicit attitudes should become increasingly important.

This study has some limitations. The first relates to the type of IAT technique used and the order effect in the first blocks because the software that was used in this study did not counterbalance those blocks. The use of the Brief-IAT technique, which does counterbalance the blocks, should be considered in future studies. The second limitation relates to the instrument used to measure explicit attitudes toward organic food. Although the final instrument used in this study had adequate psychometric properties, it only retained a small number of items from the original instrument proposed by Gil et al. [85]. Refinement of this instrument is needed to avoid potential content and convergent validity problems in future studies. The third limitation is that this study was only carried out in the United Kingdom. The United Kingdom has the sixth highest organic retail sales in the world [101] and a dynamic and growing organic market. However, the use of data from a single country prevents the generalization of results, so future research should compare countries with high and low demand for organic food. Such a comparison could help generalize the present findings. Additionally, overpricing, misgivings about the possibility of organic fraud, and the often unattractive appearance of organic food with respect to conventional food may reduce the likelihood that organic food is bought. Therefore, investigating how to overcome these and other barriers would be of great value to extend the present approach to the attitude–behavior discussion.

In summary, this study shows that different types of attitude play different roles depending on the nature of the product. Thus, differentiating between hedonic-oriented and utilitarian-oriented food products can improve the explanation of the predisposition to buy organic versus conventional food. In particular, implicit attitudes are useful to explain the predisposition to buy hedonic-oriented food, whereas explicit attitudes are effective in relation to food with a utilitarian-oriented nature. Companies can take advantage of this knowledge to design products or advertising. In addition, this study provides an understanding of how consumers perceive and are encouraged to make choices between organic and conventional products.

## Figures and Tables

**Table 1:** Sequence of implicit association test tasks used.

Rounds	Tasks	Left-Assigned Answers	Right-Assigned Answers
1	Trial of concept discrimination	Organic food	Conventional food
2	Trial of attribute discrimination	Positive	Negative
3	Trial of combined tasks	Organic food + Positive	Conventional food + Negative
4	Test of combined tasks	Organic food + Positive	Organic food + Negative
5	Trial of reversed concept discrimination	Conventional food	Organic food
6	Trial of reversed combined tasks	Conventional food + Positive	Organic food + Negative
7	Test of reversed combined tasks	Conventional food + Positive	Organic food + Negative

**Table 2:** Discriminant validity of instruments.

Factors	Cov.	SE	CI of Corr.
UTI—HED	0.24	0.04	0.15, 0.33
UTI—PRO	0.18	0.05	0.09, 0.27
UTI—PRE	0.22	0.04	0.14, 0.31
HED—PRO	0.30	0.04	0.22, 0.38
HED—PRE	0.31	0.04	0.23, 0.39
PRO—PRE	0.83	0.02	0.80, 0.86

Cov. = covariance; CI = confidence interval; Corr. = correlation; SE = standard error; UTI = utilitarian attitude; HED = hedonic attitude; PRO = attitudes toward organic food; PRE = predisposition to buy organic food.

**Table 3:** Validation of the differences between products and dimensions.

Dimension	Mean (SD)			*t* Test			Cohen’s *d*
	Milk	Chocolate		*t* Value	df	*p*	
Hedonic	4.7 9(1.29)	5.88 (0.98)	Hedonic (Milk–Chocolate)	11.24 ^a^	529.86	0.00	0.95
			Utilitarian (Milk–Chocolate)	−10.17 ^a^	555	0.00	0.85
Utilitarian	5.91 (1.07)	4.94 (1.20)	Milk (hedonic–utilitarian)	−16.15 ^b^	285	0.00	0.95
			Chocolate (hedonic–utilitarian)	14.20 ^b^	270	0.00	0.86

SD = standard deviation; t = *t* test; CI = confidence interval; Corr. = correlation; F = ANOVA test; HED = Hedonic; UTI = Utilitarian; df = degrees of freedom; *p* = probability. Cohen’s *d* > 0.8 denotes a large effect, a = *t* test for independent samples, b = *t* test for related samples.

**Table 4:** Descriptive statistics.

Variables	Range	Mean (SD)	Skewness
IA	−2, +2	0.11 (0.50)	−0.18
HED	1–7	Global: 5.32 (1.27) Chocolate: 5.88 (0.98) Milk: 4.79 (1.29)	−0.75
UTI	1–7	Global: 5.44 (1.23) Chocolate: 4.94 (1.20) Milk: 5.91 (1.07)	−0.61
PRO	1–7	4.49 (1.43)	−0.41
PRE	1–7	5.11 (1.22)	−0.23

IA = implicit attitudes; HED = hedonic dimension; UTI = utilitarian dimension; PRO = attitudes toward organic food; PRE = predisposition to buy organic food.

**Table 5:** Goodness of fit and entropy criterion for the hypothesized models.

Indicators	Independence (Model 1)	Dependence (Model 2)
Satorra–Bentler’s scaled chi-square (SBSCS)	1041.433, df = 446, *p* = 0.00	1032.65, df = 444, *p* = 0.00
Normed chi-square (NCS)	2.33	2.33
Comparative fit index (CFI)	0.93	0.93
Root mean square error of approximation (RMSEA)	0.07	0.07
Confidence interval of RMSEA	0.06–0.08	0.06–0.07

**Table 6:** Equivalence of the construct coefficients.

Dependent Variable: Predisposition to Buy		Product		*t* Test		
		Chocolate *	Milk *	*t* Value	df	*p*
Independent variables	Implicit attitudes	0.26 (0.08)	0.14 (0.08)	1.06	551	0.14
	Utilitarian dimension	−0.10 (0.06)	0.11 (0.05)	2.58	551	0.01
	Hedonic dimension	0.25 (0.06)	−0.01 (0.04)	3.60	551	0.00
	Attitudes toward organic food	0.65 (0.05)	0.54 (0.04)	1.71	551	0.04

* coefficient (robust standard error).

**Table 7:** Standardized solution.

Dependent Variable: Predisposition to Buy		Product	
		Chocolate *	Milk *
Independent variables	Implicit attitudes	0.13 *	0.07
	Utilitarian dimension	−0.09	0.11 *
	Hedonic dimension	0.23 *	−0.01
	Attitudes toward organic food *R*^2^	0.85 * 0.84	0.78 * 0.65

* *p* < 0.01.

## Appendix A

**Table A1:** Items and their basic descriptive statistics.

Constructs and Items	M (SD)	*λ*	*R* ^2^	CR	AVE
Utilitarian attitude (UTI)				0.89	0.63
UTI1. Effective/Ineffective	5.66 (1.33)	0.75	0.56		
UTI2. Helpful/Unhelpful	5.42 (1.43)	0.78	0.60		
UTI3. Functional/Not functional	5.47 (1.53)	0.86	0.74		
UTI4. Necessary/Unnecessary	5.24 (1.63)	0.76	0.58		
UTI5. Practical/Impractical	5.43 (1.44)	0.81	0.65		
Hedonic attitude (HED)				0.93	0.73
HED1. Not fun/Fun	5.24 (1.61)	0.86	0.74		
HED2. Dull/Exciting	5.14 (1.49)	0.88	0.78		
HED3. Not delightful/Delightful	5.57 (1.42)	0.85	0.72		
HED4. Not thrilling/Thrilling	4.73 (1.60)	0.80	0.64		
HED5. Enjoyable/Unenjoyable	5.92 (1.29)	0.68	0.46		
Attitudes toward organic products—OP—(PRO)				0.91	0.67
PRO1. OP are healthier.	4.90 (1.70)	0.85	0.72		
PRO2. OP are higher quality.	4.65 (1.67)	0.93	0.86		
PRO3. OP are a fraud. (R)	deleted				
PRO4. OP are tastier.	4.20 (1.61)	0.74	0.55		
PRO5. OP are worse than conventional food. (R)	deleted				
PRO6. OP are more expensive. (R)	deleted				
PRO7. OP are more attractive.	4.19 (1.63)	0.74	0.54		
PRO8. OP have no harmful effects.	deleted				
PRO9. OP are in fashion.	deleted				
Predisposition to buy organic vs. conventional food (PRE)				0.96	0.76
PRE1. Harmful/Beneficial	5.30 (1.27)	0.82	0.67		
PRE2. Foolish/Wise	4.98 (1.44)	0.86	0.75		
PRE3. Bad/Good	5.28 (1.25)	0.90	0.81		
PRE4. Unpleasant/Pleasant	5.10 (1.30)	0.84	0.70		
PRE5. Against/For	4.95 (1.50)	0.88	0.77		
PRE6. Unfavorable/Favorable	5.06 (1.48)	0.91	0.83		
PRE7. Negative/Positive	5.22 (1.40)	0.92	0.84		
PRE8. Unattractive/Attractive	5.00 (1.37)	0.83	0.69		
Implicit attitude	0.11 (0.50)				

M = mean, SD = standard deviation, λ = standardized factor loading, *R*^2^ = R-squared, CR = composite reliability, AVE = average variance extracted.
